# Dataset on stroke infarct volume in rodents: A comparison of MRI and histological methods

**DOI:** 10.1016/j.dib.2024.110188

**Published:** 2024-02-15

**Authors:** Rebecca Z. Weber, Davide Bernardoni, Nora H. Rentsch, Beatriz Achón Buil, Stefanie Halliday, Mark-Aurel Augath, Daniel Razansky, Christian Tackenberg, Ruslan Rust

**Affiliations:** a Institute for Regenerative Medicine, University of Zurich, Schlieren 8952, Switzerland; b Neuroscience Center Zurich, University of Zurich and ETH Zurich, Zurich, Switzerland; c Department of Health Sciences and Technology, ETH Zurich, Zurich, Switzerland; d Faculty of Medicine, Institute for Biomedical Engineering and Institute of Pharmacology and Toxicology, University of Zurich, Zurich 8052, Switzerland; e Department of Information Technology and Electrical Engineering, Institute for Biomedical Engineering, ETH Zurich, Zurich 8093, Switzerland; f Department of Physiology and Neuroscience, University of Southern California, Los Angeles, CA 90089, United States; g Zilkha Neurogenetic Institute, Keck School of Medicine, University of Southern California, 1501 San Pablo St., Los Angeles, CA 900893, United States

**Keywords:** Stroke volume, Ischemia, Preclinical stroke research, Stroke outcome, MRI, Histology

## Abstract

This dataset offers images of mouse brains impacted by photothrombotic stroke in the sensorimotor cortex published by Weber et al. NeuroImage (2024). Data is gathered using two primary techniques: (1) whole-brain *ex-vivo* magnetic resonance imaging (MRI) and (2) 40 µm thick coronal histological sections that undergo immunofluorescence staining with NeuroTrace. Infarct areas and volumes are assessed through MRI at two distinct time frames—three days (acute) and 28 days (chronic) following photothrombotic stroke induction. Subsequently, the brains are sectioned into 40 µm thick coronal slices, stained with NeuroTrace, and imaged as whole sections. The dataset holds considerable value for reuse, particularly for researchers focused on stroke volume estimation methods as well as those interested in comparing the efficacy of MRI and histological techniques.

Specifications Table

**Table:** 

Subject	Biological Sciences; Neuroscience: General
Specific subject area	Stroke Lesion size estimation in an experimental photothrombotic stroke model in mice.
Data format	Raw and analysed data
Type of data	Table Image Graph Figure
Data collection	Animals: Adult female C57BL/6 mice (Schlieren, ZH, Switzerland) MRI: 7T small animal scanner (Bruker, Ettlingen, Germany) Microscopy: Axio Scan Z.1 slide scanner (Carl Zeiss, Germany) Image Analysis Software: ImageJ Mathematical computation/Figure Generation: MATLAB (R2022a) Statistical analysis/Figure Generation: R-Studio (4.3.1)
Data source location	Institution: **Institute for Regenerative Medicine**, University of Zurich (UZH) City/Town/Region: Zurich, ZH Country: Switzerland (CH)
Data accessibility	Repository name: **Zenodo** Direct URL to data: https://zenodo.org/records/8056094 Data identification number: 10.5281/zenodo.8056094
Related research article	Weber RZ, Bernardoni D, Rentsch NH, Buil BA, Halliday S, Augath MA, Razansky D, Tackenberg C, Rust R. A toolkit for stroke infarct volume estimation in rodents. Neuroimage. 2024 Jan 12:120518. doi: 10.1016/j.neuroimage.2024.120518. Epub ahead of print. PMID: 38219841 [1].

## 1 Value of the Data

• The dataset offers 2D NeuroTrace-stained brain images and full brain *ex-vivo* MRI images from mouse stroke tissue at acute (3 days post injury) and chronic (28 days post injury) time points.
• This dataset contains manual lesion segmentation and automated volume estimation of ischemic brain sections from a total of 10 animals, done and validated by three experts.
• We observe lesion volumes that are highly correlated, whether using full brain MRI images or sliced NeuroTrace-stained brain sections at acute and chronic time points.
• This data can be employed by neuroscience researchers to detect and quantify ischemic lesions. Furthermore, the data enhance studies on stroke treatment and brain imaging techniques.
• With high reuse potential, the data could validate new imaging quantification methods and inform computational models.

## 2 Background

Stroke volume plays a crucial role in determining the severity of infarcts and is an important measure for assessing treatment efficacy in preclinical animal models [2], [3], [4], [5]. However, accurate estimates of stroke infarct volume can be challenging and can considerably vary between studies [6]. Several methods are suitable to investigate the extent of brain damage in experimental stroke models including magnetic presonance imaging (MRI) and histological staining. We therefore generated datasets of (1) whole brain *ex-vivo* magnetic resonance imaging (MRI) and (2) brain sections processed with immunofluorescence staining from stroked mice at acute (3 days) and chronic (28 days) time points after photothrombotic stroke to establish a semi-automated toolkit for more accurate and streamlined stroke volume estimation [1].

## 3 Data Description

The dataset [1] from the ZENODO repository includes a protocol (“**Protocol_data_postProcessing.docx”**) with step-by-step instructions on how to process MRI and histology data to estimate lesion size volume in mice subjected to photothrombotic stroke. We further provide a FIJI macro for isolating delineated lesion ROIs (“**IsolateSections_FijiMacro.jim**”) and a MATLAB code for lesion volume estimation (“**code_general_single.m”).** The code has been commented extensively to explain every command line. This should allow for smooth debugging. Please refer to the provided step-by-step protocol for further guidance.

The folder “Brains” contains all raw and processed data associated with the lesion area/volume estimation of animals subjected to a photothrombotic stroke and perfused either 3dpi (folder “acute”, *n =* 6) or 28 dpi (folder “chronic”, *n =* 4). Raw MRI data is available as -.dcm file (**MRI_dataset.dcm**) and “**NT_dataset**” contains NeuroTrace-stained brain sections (resized). The “**results_ID_MRI**” sheet shows the area values (mm^2^) of the MRI lesion ROIs; the “**results_ID_NT**” sheet contains the NeuroTrace lesion ROI areas (mm^2^). All required variables for the MATLAB script are stored in the “**referencetable**” sheet (experiment number, name of the output folder, timepoint, frame number of the scan at bregma = 0.0, bregma values for the image sequence (lowest to highest)). Delineated ROIS are stored as RoiSet (e.g. “**RoiSet_ID_MRI.zip**”) and as isolated image sequence (e.g. “**Isolated_sections_MRI.zip**”). MATLAB-exported figures are stored in an editable-.fig format and .-pdf/-.png format in the folder “**Figures**”.

The “Suppl. Table 1” sheet details all raw and analysed values from the study, including (1) estimated volume per individual animal obtained from both MRI and histology (mm^3^), (2) average ROI area measured at defined bregma positions for both timepoints, acute and chronic, and both methods, MRI and histology (mm^2^), (3) ROI area measured at pre-defined bregma positions for each individual animal, for both methods, MRI and histology (mm^2^), (4) GFAP ROI fluorescence intensities for each individual animal, (5) vascular parameters (area fraction, number of branches per mm^2^, vessel length per mm^2^) for each individual animal, (6) average cortical lesion depth per animal (%), (7) calculated dice index for MRI/histology ROI overlap and (8) between two individual observers. The “Suppl. Table 2” sheet contains all statistical data.

## 4 Experimental Design, Materials and Methods

Methods for measuring lesion volume were compared utilizing histology and MRI from the brains of mice (*n =* 10) that had undergone cortical ischemia. All procedures adhered to governmental, institutional (University of Zurich), and ARRIVE guidelines and were approved by the Veterinarian Office of the Canton of Zurich (ethics approval code: 209/2019). A total of 10 adult (12–15 weeks) female C57BL/6 mice were utilized (*n =* 6 for acute timepoint, *n =* 4 for chronic timepoint). Previous studies showed similar stroke pathology between male and female mice after photothrombotic stroke [7]. C57BL/6 mice were bred at the Laboratory Animal Services Center (LASC) in Schlieren, CH. The mice were housed in standard type II/III cages with a 12 h day/light cycle (lights on at 6:00 A.M.), and they had ad libitum access to food and water. Prior to the experiment, all mice underwent a minimum one-week acclimatization period. All animals received a large photothrombotic stroke to the right sensorimotor cortex. At 3 and 28 days after injury induction, whole heads were collected, formalin-fixed and imaged using T2-weighted MRI. Brains were removed, dissected, and histologically analyzed. We chose the timepoints based on previous literature [8], [9], [10]; animals were categorized according to the phase of stroke as acute (<3 days post-stroke) or chronic (≥28 days post-stroke).

### 4.1 Photothrombotic Stroke Induction

Anesthesia was performed using isoflurane (5% induction, 1.5–2% maintenance, Attane, Provet AG). Novalgin (1mg/ml) was applied via drinking water; 24 h prior to the procedure and for three consecutive days directly after stroke surgery. Cerebral ischemia was induced by photothrombotic stroke surgery as previously described [2,[9], [10], [11], [12], [13]]. In brief, the animals were secured in a stereotactic frame (David Kopf Instruments) and the surgical site was sanitized using betadine (Mundipharma, Germany). Next, the skull was exposed through a midline incision and a cold light source (Olympus KL 1,500LCS, 150 W, 3020 K) was positioned over the right forebrain cortex (anterior/posterior: -1.5 – +1.5 mm, medial/lateral: 0 mm to +2 mm relative to bregma). Rose Bengal (15 mg/ml, in 0.9 % NaCl, Sigma) was intraperitoneally injected 5 min before illumination, and the region of interest was illuminated through the intact skull for 10 min. The incision was closed with and the animals were allowed to recover.

### 4.2 Sample Preparation and MRI Protocol

Animals were euthanized using pentobarbital (i.p, 150 mg/kg body weight, Streuli Pharma AG) and transcardially perfused with Ringer solution (containing 5 ml/l Heparin, B. Braun) followed by paraformaldehyde (PFA, 4%, in 0.2 M phosphate buffer, pH 7). For MRI procedure, whole mouse heads were collected and post-fixed in 4% paraformaldehyde (PFA) solution for 36 h. T2-weighted images were acquired on a 7T small animal scanner with 16 cm bore size (Bruker, Ettlingen, Germany). The fixed brains were put into an Eppendorf cap filled with perfluoropolyether (Fomblin Y, Sigma-Aldrich, Switzerland) and imaged using a cryogenically cooled quadrature surface coil (Bruker, Fällanden, Switzerland). A package of 20 slices with 0.3 mm thickness (no interslice gap) was acquired with a FLASH sequence with a field of view of 15 mm x 15 mm and matrix size of 300×240, yielding a spatial in-plane resolution of 50 µm x 50 µm (echo time TE=10 ms, repetition time TR=400 ms, 10 repetitions, total scan time 10 min 40 s).

### 4.3 Histology

For immunostainings, 40 µm coronal brain sections were blocked with 5% normal donkey serum for 1 h at room temperature and incubated with NeuroTrace (1:2000, in 0.1M PB, Sigma) for 30 min. Sections were mounted using Mowiol as previously described [11,14].

### 4.4 Data Processing

Each fluorescence brain slice was registered to the corresponding *ex vivo* MRI slice using a 2D affine transformation. We used a tool provided by Fiji (Landmark Correspondences) [15]. Landmarks for registration were selected manually by a qualified researcher. Lesion area was manually delineated on each coronal brain section (MRI images and NeuroTrace-stained images) using FIJI (*ImageJ*, version 2.1.0/1.53c). Numerical values for the number of pixels in the selection were obtained, converted into mm^2^ and imported into MATLAB (R2022a, The Mathworks, Natick, MA, USA). Lesion volumes were calculated using a customized script. The *modified akima interpolation* (makima) function was used to interpolate the missing values. The area under the curve (AUC) was calculated using the trapezoidal computation rule. The volume was calculated by multiplication of the lesion area and the distance between the sections as follows:

$$\int_{a}^{b}f\left(x\right)dx\;\approx \left(b-a\right)\frac{f\left(a\right)+f\left(b\right)}{2}$$

f(x) represents the cross-sectional area at *x*.

For step-by-step guidance, please refer to our protocol (“**Protocol_data_postProcessing.docx”**). Neurotrace-stained sections were visualized using an Axio Scan Z.1 slide scanner (Carl Zeiss, Germany) with a 20x/0.8 objective lens. Each histologically processed slice could be matched with the corresponding *ex vivo* MRI slice using the mouse brain atlas.

### 4.5 Statistics

Statistical analysis was performed using R-Studio. Sample sizes were designed with adequate power according to previous studies. All data were tested for normal distribution by using the Shapiro-Wilk test. The significance of mean differences between normally distributed data (MRI vs. Histology) were tested for differences with a two-tailed paired two-sample t-test. The significance of mean differences between two different timepoints (acute vs. chronic) was tested for differences using an unpaired two-sample t-test or a one-way ANOVA with post-hoc analysis (p adjustment method = holm), in case of multiple comparisons. Variables exhibiting a skewed distribution were transformed, using natural logarithms before the tests to satisfy the prerequisite assumptions of normality. To assess the relationships between MRI-based and histology-based assessments, correlation analysis was performed using the Pearson correlation coefficient (r). Data are expressed as means ± SD, and statistical significance was defined as ∗*p <* 0.05, ∗∗*p <* 0.01, and ∗∗∗*p <* 0.001.

## Limitations

This study provides snapshots of stroke volumes at two distinct timepoints, 3- and 28-days post-stroke injury induction. The absence of intermediate data points means we cannot delineate the trajectory of stroke volume changes over time.

## Ethics Statement

All procedures were conducted in accordance with governmental, institutional (University of Zurich), and ARRIVE guidelines and had been approved by the Veterinarian Office of the Canton of Zurich (ethics approval code: 209/2019). In total, 10 adult female C57BL/6 mice were used. Breeding of C57BL/6 mice was performed at Laboratory Animal Services Center (LASC) in Schlieren, CH. All animals were housed in standard type II/III cages on a 12 h day/light cycle (6:00 A.M. lights on) with food and water ad libitum. All mice were acclimatized for at least a week to environmental conditions before set into experiment.

## CRediT authorship contribution statement

**Rebecca Z. Weber:** Writing – review & editing, Writing – original draft, Formal analysis, Data curation, Conceptualization. **Davide Bernardoni:** Software, Resources, Methodology, Formal analysis, Data curation. **Nora H. Rentsch:** Formal analysis, Data curation. **Beatriz Achón Buil:** Formal analysis, Data curation. **Stefanie Halliday:** Formal analysis, Data curation. **Mark-Aurel Augath:** Formal analysis, Data curation. **Daniel Razansky:** Writing – review & editing, Writing – original draft, Validation, Supervision, Resources, Formal analysis, Conceptualization. **Christian Tackenberg:** Writing – review & editing, Writing – original draft, Visualization, Validation, Supervision, Resources, Methodology, Investigation, Funding acquisition, Formal analysis, Data curation, Conceptualization. **Ruslan Rust:** Writing – review & editing, Writing – original draft, Visualization, Validation, Supervision, Software, Resources, Project administration, Methodology, Investigation, Funding acquisition, Formal analysis, Data curation, Conceptualization.

## Data Availability

Dataset on Infarct Volume in Rodents: A Comparison of MRI and Histological Methods (Original data) (Zenodo).

## Appendix Supplementary materials

Image, application 1

Image, application 2

Image, application 3
